# Multi-omic data integration and analysis using systems genomics approaches: methods and applications in animal production, health and welfare

**DOI:** 10.1186/s12711-016-0217-x

**Published:** 2016-04-29

**Authors:** Prashanth Suravajhala, Lisette J. A. Kogelman, Haja N. Kadarmideen

**Affiliations:** Department of Large Animal Sciences, Faculty of Health and Medical Sciences, University of Copenhagen, Grønnegårdsvej 7, 1870 Frederiksberg C, Denmark

## Abstract

In the past years, there has been a remarkable development of high-throughput omics (HTO) technologies such as genomics, epigenomics, transcriptomics, proteomics and metabolomics across all facets of biology. This has spearheaded the progress of the systems biology era, including applications on animal production and health traits. However, notwithstanding these new HTO technologies, there remains an emerging challenge in data analysis. On the one hand, different HTO technologies judged on their own merit are appropriate for the identification of disease-causing genes, biomarkers for prevention and drug targets for the treatment of diseases and for individualized genomic predictions of performance or disease risks. On the other hand, integration of multi-omic data and joint modelling and analyses are very powerful and accurate to understand the systems biology of healthy and sustainable production of animals. We present an overview of current and emerging HTO technologies each with a focus on their applications in animal and veterinary sciences before introducing an integrative systems genomics framework for analysing and integrating multi-omic data towards improved animal production, health and welfare. We conclude that there are big challenges in multi-omic data integration, modelling and systems-level analyses, particularly with the fast emerging HTO technologies. We highlight existing and emerging systems genomics approaches and discuss how they contribute to our understanding of the biology of complex traits or diseases and holistic improvement of production performance, disease resistance and welfare.

## Background

Finding causal and regulatory gene variants and using predictive genetic markers or biomarkers for complex diseases and traits constitute a major baseline for all facets of genomics, including livestock genomics. Genome-wide association studies (GWAS) have provided useful insights into the genetic architecture of complex diseases and traits in the form of potential causal single nucleotide polymorphisms (SNPs), structural variants and candidate genes. Nevertheless, there is weak evidence on how these GWAS findings improve our understanding of the molecular pathways that are involved in diseases and complex traits, thus bringing challenges to post-GWAS for the characterization of the molecular data [1]. Such association patterns are deduced from beyond the genome to the functome scale. Since two or more high-throughput omics (HTO) technologies (genomics, epigenomics, transcriptomics, proteomics, metabolomics, metagenomics and even beyond) can potentially be applied to the same animal or to biological samples from the same animal, it is important to assess how these diverse datasets at different biological levels can be integrated to exploit the full potential of such information for a holistic improvement of production performance, disease resistance and welfare in animals. It will also be interesting to see how such HTO technologies may be used in a specific omics assay [2]. The biology of systems has been meaningfully connected to today’s genetics of systems, appropriately termed ‘systems genetics’ or ‘systems genomics’ [3] in the context of animal breeding. In this process, researchers have been successful in defining the terms from ‘omes’ to the suffix ‘logy’ by positively exploiting our knowledge (Table 1). ‘Systems genetics’ or ‘systems genomics’ approaches [3, 4] study complex traits that are measured using ‘omic’ technologies such as genome/exome arrays, gene expression arrays, mass spectrometry and next-generation sequencing (NGS). These technologies are applied to study genomes, transcriptomes, epigenomes or methylomes and to link variations in these internal or ‘endo-phenotypes’ to external or ‘ecto-phenotypes’. Systems genetics discusses a branch of systems biology that integrates ‘omics’ scale data and uses them to identify causal genes and networks, regulatory genes and networks and predictive markers for complex traits.

**Table 1:** Overview of the different ‘omic’ levels used in systems genomic analyses

	Description	References
Genome	Complete collection of DNA, containing all the genetic information of an organism	[5]
Epigenome	Complete collection of changes to the DNA and histone proteins	[6]
Transcriptome	Complete collection of RNA molecules in a cell or collection of cells	[7]
Proteome	Complete collection of proteins in e.g. a cell, tissue, or organism	[8]
Metabolome	Complete collection of small-molecule chemicals (e.g. hormones) in e.g. a cell, tissue or organism	[9–11]
Microbiome	Complete collection of (genes of) microbes in the organism	[12]
Metagenome	Complete collection of genetic material contained in an environmental sample	[13]
Phenome	Complete collection of phenotypic traits, affected by genomic and/or environmental factors in an organism	[14, 15]
Functome	Complete collection of functions described by all the complementary members in living organisms	[16, 17]

Recently, a few articles reviewed systems biology and systems genetics in an animal context [18–20]. From the animal organizational level to the individual components of the systems within animals, these papers have given an overview of ‘omics’-enabled components that are characterized by vast amounts of data. Furthermore, a collection of papers presented at the symposium on systems biology in animals have attempted to bridge the gap and demonstrated that complex regulatory relationships exist among genotypes and phenotypes with an emphasis on the applicability of these integrative systems biology methods [21]. Integrating data in the multi-omic space is difficult and tedious because of the extremely large volumes of data produced across several HTO platforms, primarily from NGS machines. Secondary datasets that are generated after quality control of the raw datasets are further analysed by bioinformatics and statistical methods to create tertiary datasets (quality-controlled final datasets) that in turn form the basis of input data to systems genomics analyses. There are a number of comprehensive repositories of data obtained from genomic, transcriptomic and phenomic resources that are specific to complex traits and may explain the effect of variants (single nucleotide polymorphisms (SNPs), insertions, deletions, copy number variations (CNV)) on these traits [22]. Studies have validated several developments that aim at combining biological findings and data obtained from various resources. Recently, a complete model that estimates effects of candidate genes responsible for diseases and of those that affect interactions was conceptualized in the form of a ‘Genome <==> Phenome Superhighway’ (GPS) [23]. Genetic mapping (either linkage or association) of internal or endo-phenotypes (e.g. based on gene expression or metabolite or protein levels or quantitative trait loci (QTL) mapping [3, 4]) has generated a wealth of data and created a need for classifying, annotating, storing and analysing these data to understand their role in the genetic variation of these endo-phenotypes. More precisely, the ‘omic’ space that contains data on a trait will advance our understanding of the functions that are associated with biochemical pathways and the interactions between macro- and micromolecules [18]. However, it is necessary to carefully quality control a wide array of HTO methods that generate multi-omics datasets to remove redundant and false-positive data. With redundancy of data representing a huge threat in terms of errors in data usage, sharing data in a common information space would reduce the amount of redundant data and the potential for error. It would also contribute to harmonize to a greater degree the standardization activities across different ‘omics’ data, a critical issue in view of the integration of data from these different sources [2]. Assuming that each of these HTO datasets can be quality controlled, the next logical step would be to assess how these diverse HTO datasets that represent different biological levels could be integrated and jointly analysed to exploit their full potential for the improvement of animal production, health and welfare. With the foregoing introduction to multi-omic data integration and analysis methods, our main objectives in this mini-review are to outline current and emerging methods to generate main ‘omic’ data types and highlight some applications in animal and veterinary sciences. This paper focuses only on the main HTO data types in systems genomics (genomics, transcriptomics, epigenomics, metabolomics and proteomics). The last section of this paper re-introduces ‘systems genetics’ or ‘systems genomics’ in an emerging multi-omics context and provides perspectives on how systems genomics can be used towards improving animal production, health and welfare.

## Review

### Animal genomics

For both humans and various animal species, the study of the “whole genome” goes back to the 1990s when the focus was on the identification of genetic variants using the GWAS approach, which is based on microarrays or chips with tens of thousands of SNPs. In a GWAS, each SNP is statistically tested for significance of association with the trait/phenotype of interest. In comparison with the wide variety of available human SNP chips, the number of SNPs on an animal SNP chip is much smaller, e.g. 60 K for pig and chicken, 50 K for sheep and 777 K for cattle. In livestock genomics, many GWAS focussed on production and health traits. In 2015, Sharma et al. [24] reported an extensive review on GWAS performed in cattle, pigs, and chicken. For example, GWAS on female reproduction traits in tropically adapted beef cattle [25], feed efficiency traits in pigs [26, 27], body weight in broilers [28] and obesity and metabolic diseases using the pig as a model [29] have been conducted. The advantage of performing GWAS in livestock species over humans is the availability of related animals and subsequent knowledge about the pedigree, which greatly reduces the number of individuals needed to reach sufficient power to detect genetic variants associated with the trait of interest. Furthermore, linkage disequilibrium (LD), which is much more extensive in the genome of animals than in the human genome and depends on relatedness between animals, has a positive influence on the required sample size. Animal breeding deals with such issues by using a mixed model that accounts for the population structure/pedigree [30]. An important bioinformatics task is the annotation of the GWAS variants that explain a certain proportion of phenotypic variation and the prediction of functional properties, which serve to build ontology-based functional networks based on many databases. Many web services have been created to meet the challenge, but most of them only work within the framework of human medical research. Recently, the Functional Annotation of ANimal Genomes (FAANG) International Project Consortium was launched (http://www.faang.org/), to bring together animal scientists and sustain a steady focus on collaborations among this community. Three of four committees in this FAANG consortium address key issues on the functional annotation of animal genomes based on contributions from researchers worldwide, i.e. the Animals, Samples and Assays (ASA), Bioinformatics and Data Analysis (B&DA) and Metadata and Data Sharing (M&DS) committees. In addition, the 1000 Bull Genomes Project has provided the bovine research community with a huge volume of data on bovine variants that will be useful for GWAS and the identification of causal mutations (http://1000bullgenomes.com). These initiatives pave the way for a systematic incorporation of the findings of systems biology and systems genetics and for making them available online.

Another very important revolution in animal breeding is genomic selection (GS), a form of marker-assisted selection in which the whole genome (SNPs) is used in combination with the pedigree to predict breeding values of animals in a certain population (reviewed in [31]). GS or genomic prediction in general consists of two stages. First, the effects of SNPs are estimated using a training population for which both phenotypic and genomic data are available. Second, the effects of known SNPs are used to predict breeding values for a population for which only genomic data are available. The genomic best linear unbiased prediction (GBLUP) method uses a genomic relationship matrix (GRM) that describes the relationship between genotyped individuals using whole-genome genotyping data and after standardizing the matrix with respect to allele frequencies, it behaves like the numerator relationship matrix **A** in a regular BLUP. GBLUP provides genomic estimated breeding values (GEBV) of animals. This GBLUP version has evolved into single-step methods (ssBLUP) also known as the HBLUP method, as for example in the studies of Legarra et al. [32], Christensen et al. [33], Meuwissen et al. [31], and Koivula et al. [34] that calculate GEBV by using both the GRM and regular **A** matrix for both genotyped and non-genotyped animals. There are several GS methods, but the most widely used are those based on GBLUP and ssBLUP due to the simplicity of their assumptions and ease of computation. Bayesian methods that assume different distributional properties of SNP effects and a finite proportion of SNPs with non-zero effects are popular to identify QTL/candidate genes in a mixed model set up. GS has huge advantages for livestock genomics, since it significantly reduces the generation interval and thereby increases the response to selection. Furthermore, traits that are difficult or even impossible to measure (e.g. milk production on bulls or carcass traits on live animals) can now be genetically predicted and used to improve breeding strategies. GS is currently extensively used, for example in the pig [35] and cattle industries [36]. Recently, the expected potential of sequence-assisted selection was shown to be unrealistic, since the increase in accuracy reached with sequence-based genotyping was small compared to high-density genotyping [37]. However, increased awareness about including quantitative trait nucleotide (QTN) or loci (QTL) has raised interest, because current GS methods still ignore the functional or biological relevance of genes and their associations with QTN and QTL, and only use genomic data to build genomic relationship matrices (GRM) between genotyped and non-genotyped relatives. Attempts have been made to evaluate the relative contribution of groups of SNPs to the total genetic variance of traits, such as, intronic, exonic, intergenic, synonymous or non-synonymous variant classes, and overall, it seems that there are no significant differences between different groups of SNPs, as shown by Do et al. [38]. Bayesian methods such as Bayes Cpi and Bayes R tend to provide more accurate predictions than GBLUP-based methods when a trait is clearly affected by major QTL. The concept of the systems genomic BLUP (sgBLUP) method [4] is based on a mixture model for whole-genome-based prediction and selection. sgBLUP uses two types of SNPs, i.e. SNPs that are functionally annotated to be relevant for the specific trait in question (e.g. by GWAS and post-GWAS SNP annotation software) and SNPs called “residual SNPs” that are commonly used to build the GRM and can be used across all traits.

### Emerging technologies in genomics and epigenomics

Technology in the genomic and epigenomic fields is developing fast and provides opportunities for new ways of investigating the genome or epigenome and further implementation in animal breeding methods. With the growing power and speed of NGS, genome-wide genetic variation is now captured at the DNA sequence level at tens of millions of genomic locations. Epigenetic variation also contributes to phenotypic variation through histone modifications and DNA methylation at the gene level, which can lead to changes in or absence of the expression of genes that underlie a phenotype or a disease. The technical features of NGS are rapidly evolving, i.e. for example, restriction-site-associated DNA sequencing (RAD-Seq), which was recently applied in chickens and showed the effectiveness of NGS for animal breeding issues [39]. Such data generated by these newest technologies will contribute to the identification of (novel) causal or regulatory variants, precise genome-wide LD patterns, and insertion-deletion (InDel) markers that will be useful in molecular-based animal breeding programs. Genotype-by-sequencing (GBS) is another novel NGS technique that was developed for plant breeding, but has potential for animal breeding due to its cost-effectiveness [40, 41]. However, its potential to reach reasonable prediction accuracy and minimal bias depends on the sequencing depth and the number of individuals that are sequenced with GBS [41]. Similarly, CNV analysis using NGS has a significant impact on the study of phenotypic variation. A paradigm shift has occurred in such CNV studies with the initial global characterization being extended to in-depth studies leading to an integrated map. Although such enrichment analyses rely on good simulation and bioinformatics analyses, the results will produce a plethora of animal data that will contribute to the study of human diseases [42]. To ensure appropriate gene expression in animals, it is necessary to determine the frequency with which semi-methylated CpG islands (CGI) or sites exist in various animal tissues, which is now possible with genome-wide DNA methylation analyses. By combining different technical approaches used to produce NGS data, it is possible to analyse genome-wide methylation patterns and profiles that match at the single-nucleotide resolution [43]. One such method is reduced representation bisulfite sequencing (RRBS) [44], which is cost-effective since it allows to sequence only about 1 % of the genome by combining restriction enzymes and bisulfite sequencing. Chromatin immunoprecipitation (ChIP) coupled with high-throughput sequencing is used to integrate and efficiently identify protein-DNA binding sites in vivo. Combining ChIP-Seq data with expression data will allow us to unravel genome-wide patterns and to capture regulons and regulatory networks. Furthermore, recently it has become possible to perform ChIP-Seq with small amounts of fixed animal tissues instead of cultured cells, which will allow systematic analyses of protein–protein interactions (PPI) networks [45]. In addition, dual luminescence-based co-immunoprecipitation (DULIP) makes it possible to detect PPI with high specificity and sensitivity. The co-immunoprecipitated luciferase tags are obtained either from ChIP-Seq methods or from other assays such as DULIP and further used to support and comprehend protein function and complex biological processes [46]. With such advancements, systems-wide analyses that integrate mutation-dependent binding patterns (protein or DNA) will become feasible. It will be interesting to see how these methods improve the identification of candidate genes, drug targets and biomarkers as well as the capture of the complete genetic and epigenetic variation to accurately predict phenotypes. Currently, these high-throughput technologies offer many opportunities to better understand the complex quantitative traits and underlying (systems) biology. The remaining challenge is to overcome the difficulties in the discovery of causal genes and variants, drug targets, vaccines and biomarkers for highly complex diseases and traits of agricultural interest.

### Animal transcriptomics

Transcriptomic research investigates the expression levels of all gene transcripts in a particular cell, at a particular time, and in a particular state. Up-regulation and down-regulation of genes result in different levels of proteins and metabolites that induce phenotypic changes in the animal. Thus, a better understanding of the regulation of genes should provide insight into the biological functioning and detection of genes that are important in diseases or production traits. The most common approach to analyze expression data is to compare expression levels between two states, e.g. healthy versus diseased or high-productive versus low-productive animals, also called differential expression analysis [47]. Several studies have focussed on the detection of differentially expressed (DE) genes for various production and health traits in different species, for example adiposity in broilers [48], muscle development in cattle [49], skeletal muscle development in pigs [50] and intestinal parasite resistance in sheep [51]. Experiments on animals are done under controlled conditions and allow their dissection for the collection of various tissue samples, such as brain tissues, which is rarely possible in human studies. For example, Band et al. [52] reported a bovine genomics project that studied expression data from spleen, placenta and brain tissue for a large group of animals. Expression studies on different tissues may lead to a better understanding of the pathophysiology of health and production traits in livestock.

Besides DE analyses, transcriptomic studies analyze gene–gene interactions by using a network approach that focuses on the detection of clusters of co-regulated genes. A popular method is the weighted gene co-expression network analysis (WGCNA) [53] which is implemented in an R-package. It detects the co-expression of genes using Pearson’s correlation and calculates the topological overlap measure (TOM) that represents the number of shared neighbouring genes across gene pairs. Based on this measure, genes are clustered and the clusters are further linked to phenotypic data to reveal the important pathways that are involved in the biological background of the trait under study. Several livestock transcriptomic studies have used this approach to elucidate the genetic and biological background of health and production traits. In sheep, numerous pathways were detected in relation to muscling [54] and intestinal parasite resistance [51]. In pigs, pathways and genes that affect muscle and meat quality were identified [55] and in Hanwoo (Korean) cattle, genes related to intramuscular fat (marbling) were detected [56].

#### Emerging technologies in transcriptomics

Following the trend in human research, transcriptomic studies that are carried out on livestock species are making a shift from microarray expression data to RNA-sequencing (RNA-Seq) data and providing new opportunities to detect novel transcripts and genetic variants. With RNA-Seq, we now have the possibility of identifying and quantifying: isoforms, exon-specific expression, allele-specific expression and haplotype-specific expression. A comparison of RNA-Seq data with microarray data and its advantages are discussed in detail by Malone and Oliver [57]. Nookaew et al. [58] performed such a comparison using real expression data in *Saccharomyces cerevisiae* from both types of platforms and showed that findings based on microarray and RNA-Seq technologies were consistent. As in microarray studies, the most commonly applied method is the detection of DE genes, as was done in cattle [59–61], horses [62] and pigs [63–65]. Results of DE analyses can be used in a systems biology approach, as reported by Lee et al. [66] who integrated DE results across tissues. Several other studies have taken a step forward by using a gene co-expression network (GCN) approach using RNA-Seq. In Nellore cattle, a first study detected eight DE genes for feed efficiency [67], while a second study applied WGCNA to unravel the genetic architecture of feed efficiency [68] and showed that co-expressed (CE) genes were mainly related to insulin responses and lipid metabolism. These findings combined with data from histopathological analyses of the liver revealed that low feed efficient animals had a larger number of liver lesions than high feed efficient animals. A similar project is being conducted using different cattle breeds (Holstein and Jersey cows) with extreme feed efficiency phenotypes [69]. A gene co-expression network (GCN) approach was applied to pigs to detect co-expressed clusters of genes related to, for example, backfat androstenone phenotype [70], *Salmonella* shedding [71] and obesity-related genes [72].

Animal reproduction through assisted reproductive technologies, such as in vitro production of embryos (IVEP) combined with genomic selection, can result in rapid genetic improvement [73]. Transcriptomic and systems biology investigations on oocytes and embryo traits related to IVP, embryo transfer and subsequent pregnancy rates have detected biomarkers for successful IVP, embryo transfer and pregnancy rates [74–76].

In pig production, one of the challenges is to reduce boar taint i.e. an offensive taste or odour of the pork emitted during cooking, which makes it unpleasant for consumers. It is caused primarily by two compounds i.e. androstenone produced in the testicles and skatole produced in the hindgut. Both compounds accumulate mainly in the back fat of intact males and the only way to reduce boar taint is by surgical castration. However, surgical castration raises serious pig welfare issues that contributed to a voluntary ban on surgical castration in Europe to avoid boar taint (http://boars2018.com/). Heritabilities of androstenone and skatole levels are moderate to high [77], which indicates that breeding for low boar taint in males has good potential and may resolve welfare issues in the long-run, since intact males could then be used in the food chain. GWAS has detected several genomic regions associated with androstenone and skatole levels [77–79]. It was shown that boar taint is not significantly correlated with growth traits and litter size [80, 81] and is even favourably correlated with male fertility [81]. Recent studies on RNA-Seq transcriptome profiling of pigs with high or low boar taint showed that there are key differences in the expression profiles of some genes [70, 82]. Following this, our studies now focus on RNA-Seq transcriptomics and systems biology of boar taint in Danish pigs, to identify DE and CE genes and build GCN to improve pig meat production in Denmark [83]. To gain more insight into the regulatory architecture of a particular trait or disease, several approaches can be combined or integrated to elucidate gene–gene interactions.

Another relatively novel and promising method is single-cell transcriptome analysis, giving a deep-sequencing insight into the cell’s gene transcription [84]. This deeper insight can lead to a better understanding of the link between genome and phenome studies, and has potential mainly in cell development research (e.g. stem cell research) and in situations where the collection of biopsy samples in sufficient numbers for RNA-Seq transcriptomics is difficult (e.g. in the case of embryo biopsies before embryo transfer to donor cattle). Although a wide range of candidate genes and transcripts using RNA-Seq analyses can be exploited, the major challenge is to identify true positives. Methods based on mapping and genome assemblies might miss some candidate genes, if appropriate filtering techniques are not used. Another challenge is to quantify G/C blocks, paralogons, isochores, 5′UTR regions, expression specific to splice variants, exon-specific and allele-specific expression. Transcriptomics studies based on RNA-Seq enable the study of non-coding RNAs but how such studies on non-coding RNAs can be used for systems genomics approaches remains to be explored. In the last few years, there have been increasing efforts to sequence small RNAs including miRNAs. Since the initial application of RNA-Seq, quantifying small RNAs, ascertaining alternate splicing events, transcription start sites (TSS) and mapping strand-specific genes [85] have benefited from such techniques. Many studies are underway to provide rigorous strategies for miRNA-Seq and other small RNA measurements, but this is beyond the scope of this review.

### Animal metabolomics and proteomics

Proteomics aims at describing the complete repertoire of proteins in an organism [8], while metabolomics (or metabonomics) is the study of global metabolite profiles in living systems [11]. Although the use of both terms metabolomics and metabonomics is still debated, analysis of the metabolome is a challenging task since it considers all the metabolites, regardless of their chemical nature, i.e. amino acids, antibodies, aptamers, small biomolecules, etc. and provides coherent gene expression data in an integrated manner. Metabolomics serves not only as a source of qualitative but also quantitative data on intracellular metabolites that are essential for the model-based description of the metabolic network operating under in vivo conditions. In recent years, several studies in livestock have investigated the metabolome, and metabolite profiling studies are now a rapidly expanding area in animal and veterinary genomics. Metabolomics tools aim at filling the gap between genotype and phenotype by permitting the simultaneous monitoring of molecules in a living system. Such metabolic information has applications in clinical practice, in the discovery of biomarkers that are linked to cellular integrity, cell and tissue homeostasis resulting from cell damage or death [86], and in metabolic engineering to optimize microorganisms for biotechnology.

In dairy cattle, numerous potential biomarkers were detected for milk production and quality by studying the metabolome of different body fluids [87]. Likewise, in chickens, several potential biomarkers were identified for the ascites syndrome by investigating the liver metabolome [88]. Another potential of metabolomics is the prediction of phenotypes that are of economic interest, as was reported for pigs [89].

#### Emerging technologies in proteomics and metabolomics

Proteomic and metabolomic datasets provide vast amounts of multi-dimensional data points that need to be carefully quality-controlled, analysed and interpreted. As in genomics and transcriptomics, there are a wide variety of publicly available databases and tools for storing, querying, browsing, analysing and visualizing metabolomic networks. For example, the PathCase Metabolomics Analysis Workbench (PathCaseMAW: http://nashua.case.edu/PathwaysMAW/Web/) runs on a manually created generic mammalian metabolic network. The mapping of protein–protein interactions (PPI) networks to phenotype and disease pathways is a key to understanding various biological and patho-physiological processes. Such interaction studies can be combined with studies on the conservation of non-coding RNAs across large evolutionary distances and on their potential functions in mammalian genomes i.e. [90]. Overall, ecosystems that are revealed by such association networks, assemblies and interaction studies are challenged by various environmental conditions. In this process, a new field has emerged termed “synecology” that deals with the interactions of groups of organisms with their abiotic and biotic environments and is driven by the advances of the meta-omics methods using bioinformatics-centred approaches [17]. This could lead to a new “omics” term i.e. synecomics defined as molecular systems synecology, which will contribute to understand not only the mammalian or animal dynamics, but also the microbial processes that rely on systems-level responses.

Recently, many structural proteomics initiatives were launched to ascertain biochemical and cellular functions and have allowed the design of drugs at the molecular level [91]. The methods used include advances in hardware design, data acquisition methods, sample preparation and further automation of data analysis. With 40 to 50 % of the identified genes corresponding to proteins of unknown function, a functional annotation screening technology using nuclear magnetic resonance (NMR) (FAST-NMR) was developed to assign a biological function. These methods assume that a biological function can be described based on the similarities between binding regions among proteins and that a given ligand interacts with a targeted sequence. The resulting structural and functional assignment to a protein can provide a starting point for the discovery of drugs as well as functional clues for regions that are regulatory or non-regulatory [91]. Nevertheless, functional proteomics/metabolomics has evolved as the necessary next step for which NMR spectroscopy is used to study the functions of a large repertoire of sequences that cannot be inferred based on the current methods for the detection of sequence homologies alone. Moreover, three-dimensional structures of proteins/metabolites contribute greatly to inferring molecular functions (physical and chemical function). We can foresee that systems genomics in the future will embrace large-scale proteomics/metabolomics as an additional layer to provide connections with phenotypic variations.

### Functional annotation and pathway analyses

In the preceding sections, we discuss individual ‘omics’ platforms and datasets with regards to their current status and emerging trends. Regardless of which ‘omics’ platforms are used, the Gene Ontology (GO) annotation is the most important and valuable means of assigning functional information using standardized vocabulary. Several computational methods and tools are available for functional annotation across all species. Gene-based annotation can identify whether SNPs or CNV cause protein coding changes. For this purpose, gene definition systems such as RefSeq genes, UCSC genes, ENSEMBL genes, GENCODE genes are used. Genomic region-based annotation identifies variants in specific genomic regions, for example, conserved regions, NGS-based DE/CE regions, transcription factor binding sites, GWAS regions, etc. Although similarity-based GO annotation is widely applied, it primarily encompasses sequence data with reciprocal best hits to predict candidates from a huge repertoire of multi-omics data. However, some of the orthologues of these sequences do not remain associated to GO terms and can be cross-validated with conserved domains, manually reviewed data or determined by wet lab experiments, thus allowing the biological appropriateness of the functional assignments. The unannotated regions in the form of hypothetical proteins or “known unknowns” i.e. their existence is predicted but their function is not known, represent a huge problem, since they remain assigned to the three root terms as in the case of AMIGO (http://amigo.geneontology.org/amigo). A few methods have been designed to integrate different structural and functional results with data corresponding to GO relationships of organisms [92]. In addition, the genome assemblies of many species are regularly refined and updated when new information is available. There has been an increase in the development of integrated analyses that provide comprehensive and robust GO annotations of genome assemblies, providing a solid foundation for functional interrogation of other genomes (http://www.ebi.ac.uk/GOA). Development of pathway maps and identification of unique and novel signals have transformed pathway association studies in cattle [93]. Furthermore, Medical Subject Headings (MeSH, http://www.nlm.nih.gov/mesh) provides a comprehensive life science vocabulary for human and model organisms’ research. Multi-faceted ‘omics’ is aided by the choice of annotation and enrichment analyses for interpreting GO-aided MeSH functional terms. In summary, such GO annotations correspond to specific biological conditions or complex traits in specific species.

Pathway analysis can be described as “a group of statistical methods that exploit a priori knowledge of pathways” [94]. It forms the link between ‘omics’ results and the phenotype/disease under study and provides a biological meaning to the genes and variants detected (interpretation of results). Furthermore, it reduces the multiple-testing burden and, thus, offers a huge analysis potential. Aslibekyan et al. [94] showed that in spite of the great potential of pathway analysis, there are still many obstacles to overcome (for example, due to the lack of a golden analysis standard).

#### Emerging technologies in pathway profiling and genetic networks

In spite of the multidimensional HTO efforts to understand phenotypic variation, there remains a major scientific bottleneck regarding the inference of contextual pathways that underlie the translation from variation in biological systems to phenotypic variation. Recently, some integration models that address the characterization of the interaction between functional modules have been reported [95]. For example, the PAthway Network Analysis approach (PANA) integrates high-throughput data and their functional annotation using machine-learning methods [96]. The end-user can detect the functional modules that are associated within the molecular system and the transcriptional connections in a disease or a phenotype. Molecular systems biology integrates networks in the form of pathways, interactions and/or associations. Associations are inferred only as links within the relationships, whereas physical relationships in the form of pull-down assays or biochemical experiments are inferred as interactions. Nevertheless, the paradigm that all interactions are associations but not all associations are interactions can be widely applied across all functional modules. As discussed previously, keeping in view the various biochemical pathways and reactions, the models aim at analysing and measuring the quantity of molecules that are present within a cell [20]. However, the models used depend on the type of assay, on how epigenetic modifications are deduced from the transcriptomic data, on whether or not the disease risk is considered, and on the type of genetic heterogeneity investigated in relation to the phenotypic trait [97, 98].

Many studies have shown the importance of genetic interactions, especially in the determination of complex polygenic traits [99–101], which support the great potential for network genetics. In general, a GWAS investigates the genome of different individuals to detect variants that are associated with a trait, but it does not take the interactions between loci into account. However, studies on genome-wide interactions among SNPs for specific traits are being carried out, see for example, a paper on carcass-related traits in Brahman cattle [102]. One further step is to include epistatic interactions in a network approach, for example using the weighted interaction SNP hub (WISH) network method [103], which has been successfully applied to a pig resource population to detect genes and pathways related to human obesity [29]. This method pre-selects SNPs based on their genome-wide significance by setting a much lower significance level than in standard GWAS, and subsequent calculation of the epistatic interaction effects between all SNPs is used in a clustering approach. Another promising method is the association weight matrix (AWM) approach, which combines data from several GWAS by looking at interactions between SNPs based on the sizes of their estimated additive effect [104]. This method has successfully identified genes and pathways for growth in cattle [105] and puberty in tropical cattle breeds [106]. The latest reports show how lncRNAs contribute to regulatory interactions with their non-coding peers such as miRNAs [107]. Whether lncRNA-protein networks restrain interactions is not clearly known. How such regulatory interactions between classes of lncRNAs and proteins can have a significant influence on an organism is a focus of interest. Recently, our group reported the detection of one such lncRNA-protein association that was consistent with interaction networks built from RNA-Seq data [108]. These studies will allow us to understand how such association networks contribute to transcriptional regulation in various organelles. In addition, applying network methods and pathway analyses to genes that are related to a wide range of diseases and phenotypes will allow researchers to gain deeper insight into pathophysiological and biological processes.

### Multi-omic data from genome to phenome: integration in systems genomics

The term ‘systems genetics’ or ‘systems genomics’ in an animal breeding context was originally proposed by Kadarmideen et al. [3], but there are recent reviews on this topic with applications in humans [109] as well as in animals [4]. As thoroughly discussed in these articles, systems genetics/genomics focuses on the integration of different ‘omics’ levels. This includes a wide range of approaches, from relating the individual’s ‘omics’ levels with functional annotation, both on a single gene level and pathway analysis level, to integrating all different multi-omic levels to phenotypes. A typical data integration process goes from genome → epigenome → transcriptome → metabolome → proteome → phenotype or disease variome.

The integration of data related to protein abundance/mRNA expression using regulatory networks has been investigated with respect to gene expression involved in bovine puberty [110]. Integration of genomic and transcriptomic data, for example using the expression quantitative trait loci (eQTL) approach, detects regions in the genome that are associated with transcript levels [111]. These eQTL can be cis- or trans-acting: a cis-acting eQTL is located near the gene that encodes the transcript, while a trans-acting eQTL is located at quite a distance or even on another chromosome. Several studies in pig have incorporated this approach to detect candidate genes, for example for muscle characteristics [112–114] and obesity phenotypes [115]. A recent multi-parental population study on heterogeneous stocks (HS) in rats, mice and humans identified targeted candidate genes and mapped them to disease phenotypes [116]. In this work, the authors applied differential expression analysis followed by eQTL analysis, and then using a mixed-model analysis based on the sequence of the founder animal, they identified variants within the detected region. Although such studies can detect variants within disease-causing genes, whether or not these causal genes alone may play a role in these complex phenotypes remains a challenge. Questions remain such as: (1) how important these eQTL are for the study of genetic networks that underlie phenotypic variation? (2) Can these eQTL data generated from transcriptomics analyses be linked to the proteomics level? Although transcriptomic and phenomic data may appear uncorrelated, mapping genetic determinants of gene expression (eQTL) can provide a remarkable framework for understanding large phenotypic effects and linking genetic variants to disease [117].

Clustered regularly interspaced short palindromic repeats (abbreviated as CRISPR) in combination with Cas9 protein (CRISPR/Cas) systems guide RNAs into a cell’s genome (the nuclease) and cut the genome at desired locations (this technique is often referred to as genome-editing). Since it was first introduced by Cong et al. [118], many site-directed mutagenesis experiments have been carried out across various tissues, large animal models and populations [119, 120]. This site-directed genome editing technology has extensively improved the precision at which genome modifications can be obtained compared to earlier transcription activator-like effector nucleases and zinc-finger nucleases systems. To date, it has not been used extensively in animal and veterinary sciences, but it is clearly foreseen that animal genome modification using CRISPR/Cas systems will play a key role in improving disease resistance or trait performances in animals or to create “designer animals” such as transgenic animals. One other application is that once a causal gene/QTL is validated, it can be specifically edited by applying the CRISPR-Cas system. Such modifications of the genetic architecture of an organism are only just beginning and have not been adequately studied in animals. A recent paper on human albumin produced in pigs through CRISPR/Cas9-mediated knockin of human cDNA into the swine albumin locus at the zygotic level illustrates the potential of this technology for animal research [121]. The use of RNA-Seq transcriptomic studies to infer CRISPR-mediated systems should play a crucial role in advancing this technology even further.

Animal systems genomics is still far from fully exploiting the power of deep NGS. For instance, RNAseq can provide not only accurate measures of gene expression levels but also data on isoforms, exon-specific expression, allele-specific expression and haplotype-specific expression. The benefits of RNA-Seq in systems genomics studies are yet to be fully exploited, for instance, in eQTL studies. Wang et al. [122] reported the use of the Lyon hypertensive (LH) rat bred for high blood pressure in eQTL mapping by taking advantage of RNA-Seq data. To what extent these additional benefits of RNA-Seq can be integrated in eQTL studies are still unknown but we can foresee the emergence of eQTL studies that will use this information in integrative systems genomics studies.

During the last decade, strategies to detect metabolic QTL have emerged and are based on the characterization of metabolites and small molecules using large-scale analytical methods. Such methods allow researchers to better understand the biochemical pathways that span a metabolic network. Whether or not the environment has an impact on the metabolism can be analyzed by using a phenotypic state of the metabolism called a “metabotype”. This metabolomic/metabotype quantitative trait locus (mQTL) mapping and metabolomic genome-wide association studies (mGWAS) have been widely applied to derive information from genetic polymorphism studies, see for example, a human study of cardiovascular diseases [123]. However, a comprehensive framework to understand this multi-omic convergence of high-resolution metabolomics is lacking such as that developed in the R-package mQTL.NMR for an integrative analytical framework for genomic and metabolomic profiles to characterise mixed systems [124]. The principle of mQTL or mGWAS can be also applied to a list of known metabolites (e.g. low-throughput metabolite profiles consisting of up to 200 compounds) that are assumed to affect a given disease phenotype in animals. For instance, Pant et al. [125] identified several QTL that influence a large range of metabolites affecting obesity and obesity-related phenotypes via combined linkage disequilibrium linkage analysis (LDLA) in F2 crossbred pigs, and subsequently, investigated the human chromosomal regions that were syntenic to these identified mQTL. Further discussion on this work is beyond the scope of this review, but clearly quality control and analyses of large-scale metabolic/metabolomic phenotype data represent a big challenge for animal genetic studies [126, 127]. Another novel area of research is the mapping of genetic variants that affect protein abundance (pQTL), which to date has been successfully applied on an F2 mouse population [128] and for the analysis of cellular responses to chemotherapy [129]. Such developments provide great opportunities to identify biomarkers for animal disease and production traits. Regardless of what type of QTL or SNP is detected for animal traits (eQTL, mQTL, pQTL), they can be incorporated into models that aim at understanding/detecting causal and regulatory loci in the genome, as discussed in [109] and [4].

**Fig. 1:**
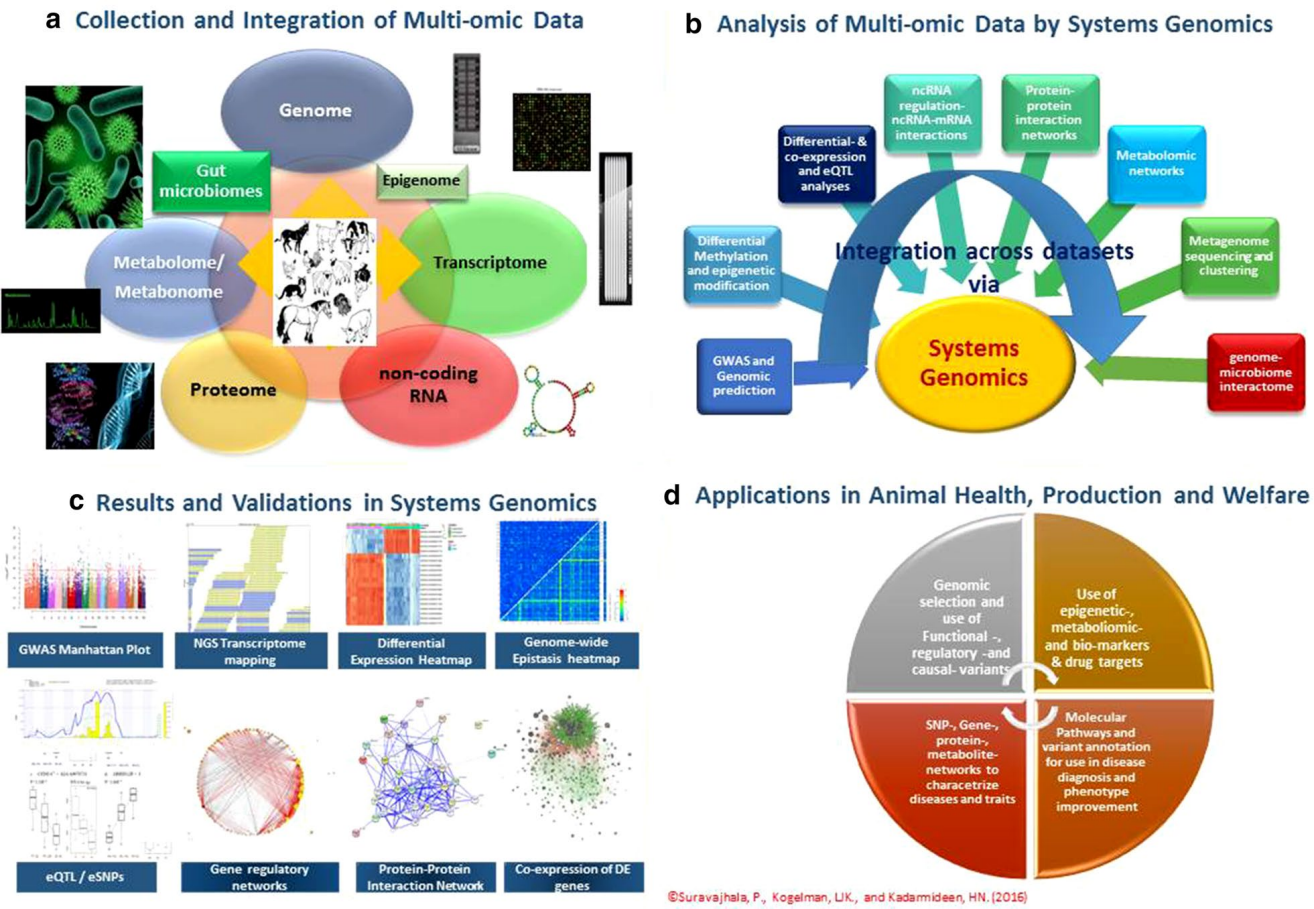
Overview of integrated genomics with various other ‘omics’ platforms/data types created via array-based or spectrometry or NGS technologies and systems genomics analyses. **a** Collection of multiple types of ‘omics’ datasets in farm or companion animals in controlled experimental conditions or in field experiments. **b** Systems genomics involves analysis of single-layer (*vertical arrows*) and multi-layer ‘omic’ datasets (*horizontal arrow*) ranging from GWAS, differential expression or methylation analyses through proteomic/metabolomic datasets to eQTL/mQTL/pQTL and network analyses. **c** Typical results of systems genomics involve single- and multi-layer analyses from GWAS Manhattan plots, genome-wide epistatic heat plots, variant detection or transcript counts in NGS data and gene expression heat plots through eQTL maps, gene regulatory or co-expression networks of eQTL or protein–protein interaction (PPI) networks to networks of differentially connected genes. The eSNP/eQTL box plot is taken from Kogelman et al. [115]. The remaining images in this panel are from the authors’ own unpublished material. **d** Potential applications of such approaches involve identification of causal genes or pathways, biomarkers, drug targets, various networks for a specific trait level or disease state and individualized genomic predictions of performance or disease risk

Figure 1 summarizes the preceding discussions on multi-omic data integration and systems genomics. It illustrates how multi-omic data are generated and integrated by various HTO platforms from the genome level through various ‘omes’ to the animal phenotype or disease variome databases. It shows individual, single-layer ‘omic’ analyses as well as multi-layer ‘omic’ analyses in a systems genomics framework. Typical results from single-layer genomic or transcriptomic analyses can be visualized in terms of GWAS—Manhattan plots, variant calling plots from NGS data, transcript counts from RNA-Seq transcriptome data, genome-wide epistasis heat maps, and differential expression heat maps, etc. With a given HTO dataset from one of the ‘omics’ platforms, single-layer specific analyses are carried out before merging a given HTO dataset with another HTO dataset. For instance, GWAS and differential or co-expression (DE/CE) analyses are conducted independently from each other before combining these HTO datasets to identify eQTL or eSNPs. This also applies to metabolite or metabolomic profiles (NMR peaks) that are affected by QTL or SNPs (mQTL or mSNPs). Conducting an epigenetic study to identify differentially methylated regions and integrating these analyses and results with transcriptome analyses that are focused on DE/CE analyses is another example. Typical results from two- or multi-omic analyses include eQTL maps, eSNP effects, mQTL or mSNP effects, directed gene regulatory networks (using SNP data), PPI networks built by using multi-omic data as evidence, co-expression networks of genes that are mapped as cis- and trans-acting eQTL or those that are differentially expressed. Applications of these multi-omic data analyses include genomic prediction/selection, use of functional, regulatory and causal variants in developing highly accurate assays for disease prevention and diagnosis or holistic trait improvement. The use of epigenetic markers, metabolomic profiles or fingerprints, biomarkers and drug targets is highly appropriate for disease prognosis/diagnosis/treatments and clinical interventions in veterinary medicine and thus contributes to improving animal health and welfare. Molecular pathways and annotation of variants can be used in disease diagnosis and phenotype improvement. Systems genomics is typically characterized by several types of interaction or association networks and includes SNP-SNP, gene–gene, protein–protein, metabolite-metabolite networks that characterize a particular disease or healthy state or performance levels in animals. These networks provide deeper insights into interactions within and across tissues as well information on master regulatory genes or variants or metabolites or proteins (the ‘hubs’) that can be used as predictive markers in disease diagnosis and phenotype improvement.

## Conclusions

In this review, we discuss the current and emerging technologies within the fields of genomics/epigenomics, transcriptomics, metabolomics and proteomics, and provide some examples from livestock species. We re-introduce systems genetics/genomics in a “sequence-space” and multi-omic context with a focus on animal and veterinary biosciences. Due to the enormous progress in (e.g. sequencing) technologies, data generation is becoming cheaper and easier, resulting in huge amounts of data at different ‘omics’ levels. In the last few decades, data were generated to elucidate the biological mechanisms that underlie animal production, health and welfare traits. This has led to great insight into mechanisms and detection of (potential) biomarkers and vaccines, and improved animal breeding strategies. We briefly mention different existing and emerging ‘omic’ technologies and their implementation in livestock species (including genomics, epigenomics, transcriptomics, metabolomics and proteomics). The challenge that remains is to use all these ‘omics’-level data sets efficiently by removing errors/noise via good quality control methods for each layer of dataset, appropriate data integration as per the defined systems genomics hypothesis and statistical models, application of advanced statistical-bioinformatic algorithms and meaningful interpretation of results. The clear advantage of these integrative methods is to increase the power of detecting true causal genes, regulatory networks and pathways leading to improved animal health, welfare and/or production. Outcomes such as causal genes or variants (QTN or QTL), regulator genes, biomarkers and gene networks should be incorporated into genomic selection and breeding programs for larger impact. These prospects are becoming more feasible, as genomic selection methods tend more and more to include various types of QTL information in genomic prediction models. Through such extended, biologically and functionally meaningful and accurate genomic selection methods, improvement of animal production, health and welfare will be even faster and more sustainable.
